# Structural ableism in public health and healthcare: a definition and conceptual framework

**DOI:** 10.1016/j.lana.2023.100650

**Published:** 2023-12-18

**Authors:** Dielle J. Lundberg, Jessica A. Chen

**Affiliations:** a Department of Health Systems and Population Health, University of Washington School of Public Health, Seattle, WA, USA; b Department of Global Health, Boston University School of Public Health, Boston, MA, USA; c Health Services Research & Development (HSR&D) Center of Innovation for Veteran-Centered and Value-Driven Care, Veterans Affairs (VA) Puget Sound Health Care System, Seattle, WA, USA; d Department of Psychiatry and Behavioral Sciences, University of Washington, Seattle, WA, USA

**Keywords:** Structural ableism, Ableism, Disablism, Disability, Neurodivergence, Chronic illness, Mental illness, Madness, Structural determinants of health, Health equity

Structural ableism has received limited attention in the public health and health services literature as a determinant of health outcomes and disparities.^1^^,^^2^ This is notable for several reasons. First, disabled people represent an estimated 16% of the world's population,^3^ and a sizable portion of the healthcare system exists to provide services to disabled people. For example, in the United States (U.S.), 36% of healthcare expenditures are related to disability.^4^ Second, interpersonal disability-based discrimination remains prevalent.^5^ In the healthcare system, explicit and implicit biases toward disability are pervasive, suggesting this is an important sector to examine the effects of structural ableism.^6^^,^^7^ Third, significant health inequities exist for and among disabled people which relate at least in part to discrimination, inaccessibility, and other barriers that disabled people experience throughout health systems and society.^8^, ^9^, ^10^ To-date, structural ableism has received greater attention in other fields, such as in prior literature by disability studies and disability justice scholars.

In this viewpoint, we offer a definition and conceptual framework for examining structural ableism in public health and healthcare and suggest potential pathways by which structural ableism may influence health. We also propose a series of principles to guide researchers in studying structural ableism and health. Throughout this article, we use identify-first (i.e., disabled person) rather than person-first language (i.e., person with a disability). We discuss our language choices in Section A of the Supplementary Material. Describing the effects of structural ableism on health greatly exceeds the scope of a single publication. For this reason, our broader goal is to inspire dialogue about why structural ableism has often been ignored in the public health and health services literature and the ways that these fields have been complicit in perpetuating and legitimizing ableism. This exploration is particularly timely given the National Institutes of Health (NIH)'s designation in September 2023 that people with disabilities are a population with health disparities and related calls for research on ableism.

## Defining structural ableism

Structural ableism is a system of historical and contemporary policies, institutions, and societal norms and practices that devalue and disadvantage people who are disabled, neurodivergent, chronically ill, mad, and/or living with mental illness and privilege people who are positioned as able-bodied and able-minded.^11^, ^12^, ^13^, ^14^, ^15^ This system impacts disabled people regardless of whether they identify as disabled and if their disability is visible and may also affect other populations perceived as deviating from social, cultural, and economic constructs of productivity and normativity.^16^, ^17^, ^18^ Structural ableism denies disabled communities equitable access to social resources and to disability competent and affirming health services, control over whether their experiences are listened to and believed, autonomy over how their needs are represented and responded to, and justice when they are exposed to harm, discrimination, and violence.^1^^,^^2^^,^^8^, ^9^, ^10^

Structural ableism is upheld via interlocking systems of power and oppression, such as racism, sexism, transphobia, capitalism, and colonialism, and operates alongside audism, sanism, and other types of disability-related bias and discrimination including internalized and interpersonal ableism.^14^^,^^16^, ^17^, ^18^, ^19^, ^20^, ^21^, ^22^, ^23^, ^24^ Within and outside of health systems, structural ableism may influence health and contribute to health inequities for disabled people through multiple pathways. Structural ableism may particularly harm disabled people who live at the intersection of multiple systems of oppression, such as those organized around race, ethnicity, socioeconomic status, gender, sexual orientation, religious affiliation and religiosity, body shape and size, age, migrant status, nationality, and/or geographic location.^19^, ^20^, ^21^, ^22^, ^23^, ^24^

This definition represents our attempt at bringing together concepts from disability studies and disability justice scholarship and situating them in the burgeoning public health and health services literature on social and structural determinants of health. This includes referencing prior work on ableism by Subini Ancy Annamma,^21^ Patty Berne,^22^ Nicole Brown,^15^ Carli Friedman,^11^ Talila Lewis,^16^ Mia Mingus,^20^ and Sins Invalid^22^ and on structural determinants of health by Zinzi Bailey,^19^ Tyson Brown,^23^ and Patricia Homan.^23^ Definitions of structural ableism should be refined over time and adapted for various contexts and communities. For example, structural audism is a related system that impacts deaf and hard of hearing communities.^24^ Section B of the Supplementary Material describes our approach to generating this definition through narrative review, and Supplementary Table S1 captures prior definitions identified in the review.

## Pathways connecting structural ableism to health

**Fig. 1:**
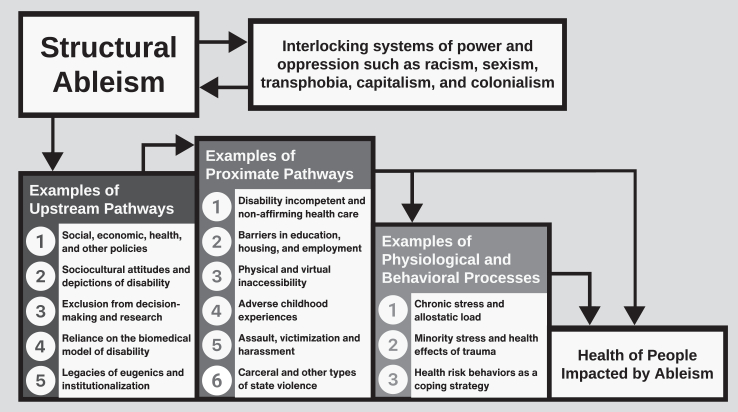
A conceptual framework highlighting examples of pathways and processes that may connect structural ableism to health outcomes for and among disabled people and other populations impacted by ableism.

Fig. 1 presents a conceptual framework describing examples of potential upstream pathways, proximate pathways, and physiological and behavioral processes through which structural ableism may influence health and contribute to health inequities for and among disabled people and other populations impacted by ableism. This framework is not comprehensive but serves to highlight the pervasiveness of structural ableism in public health and healthcare and how further research is needed to understand how structural ableism may operate in distinct ways for different disabled communities.

### Potential upstream pathways

#### Policies

Social, economic, health, and other policies (or a lack of equitable policies) shape the experiences of people who are disabled, neurodivergent, chronically ill, mad, and/or living with mental illness.^9^^,^^13^^,^^14^ Supplementary Table S2 presents a list of relevant policies and selected related scholarship. One example is the U.S., where state and federal laws dictate what health services disabled people can access and how much they pay for them. Policies can affect many aspects of life for disabled people by contributing to extra costs for health care, personal assistance services, mobility aids, and other assistive technologies. For example, disabled people are estimated to have at least twice the amount of out-of-pocket health care expenses and three times greater odds of delaying or forgoing care due to cost compared to non-disabled people.^25^ Rules within Medicaid, Supplemental Security Income, and other programs can also limit disabled people's ability to accumulate financial assets and marry without losing needed health services and benefits.^9^ State and federal laws also allow some disabled people to be paid subminimum wages and determine whether disabled people and their family members have access to paid medical and family leave. On a global scale, inclusion (or lack of consideration) of disabled people in policies related to education, social programs, health care access, political participation, linguistic development, communication access, sexual and reproductive health, war and conflict settings, and public health emergencies such as the COVID-19 pandemic are additional examples of how structural ableism may operate through policy to drive health inequities.^3^

#### Sociocultural attitudes and depictions

Sociocultural attitudes toward disabled people and depictions of disability in media, health communications, and health systems can also alter how disability is understood throughout society and lead to institutional, interpersonal, and internalized forms of ableism.^7^^,^^17^^,^^18^ When disabled people are not involved in creating societal narratives about disability, stereotypes and misinformation often proliferate instead.^26^ Such depictions often portray disabled people as villains who warrant fear, as victims who lack agency and deserve pity, and/or as heroes whose life purpose is to triumph over their disabilities.^26^ Sociocultural attitudes and depictions can shape how health professionals, researchers, and policy-makers approach disability and how families, community members, and caregivers act towards disabled people.

#### Health decision-making and research

Disabled communities encounter additional systemic exclusion when it comes to decision-making in public health and healthcare. In the U.S., a national survey found that only 3.1% of physicians report that they have a disability,^27^ and the percentage of grant applications to NIH with a principal investigator disclosing a disability was only 1.9% in 2008 and fell to 1.2% in 2018.^28^ This is significant because physicians and researchers contribute to the development of health policies and are often selected for decision-making roles within health systems. The under-representation of disabled people within these fields relates in part to experiences of educational and workplace discrimination and inaccessibility that affect retention and advancement.^29^, ^30^, ^31^, ^32^ Additionally, disabled people are largely prohibited from participating in clinical research due to the inaccessibility of study designs and unjustified eligibility criteria that restrict their engagement.^33^ Such exclusions may lead to misrepresentations about the needs of disabled people and contribute to the erasure of disability in health policy and resource allocation.^8^^,^^17^^,^^34^

#### Biomedical model of disability

The biomedical model of disability—which understands disability as impairment resulting from a health condition—has also contributed to health systems that view disability as a problem to be solved, discourage positive disability identity development, and fail to appreciate the contributions of disabled people.^13^^,^^14^ While investments in medical interventions for disability may be desired in many cases, many disabled people also report that they would choose to be disabled even if given the opportunity to be “cured”.^12^^,^^13^ For this reason, investments in programs and social conditions that improve disabled people's quality of life and reduce barriers to activity are often more appropriate and beneficial.^1^ Furthermore, the assumed objectivity and universality of the biomedical model of disability has led health professionals and researchers to rely on tools and metrics that often misunderstand how disabled people view their experiences.^34^ The social model of disability—which argues that social attitudes, discrimination, and inaccessibility make a health condition disabling and that society often creates disability through ableism—can offer a useful counterpoint to the biomedical model.^12^^,^^13^ However, scholars have noted that efforts to globalize social and human rights models of disability in place of the biomedical model should also be questioned because they can contribute to the erasure of Indigenous knowledge and reinforce colonial patterns of thinking that ignore how disability is understood and experienced in varied ways across social and cultural contexts.^35^, ^36^, ^37^

#### Eugenics and institutionalization

Finally, histories of eugenics and forced sterilization, social and educational segregation, and institutionalization rooted in ableism, racism, classism, and related systems inform health policies today that can deny disabled people their personal autonomy.^38^ In the U.S., more than 30 states enacted laws that required sterilization as a condition of release from psychiatric and other institutions during the 20th century.^38^ Supplementary Table S3 presents a list of selected scholarship examining the legacies of eugenics and institutionalization in health policy, especially for Black, Indigenous, and disabled people of color. Today, some disabled people continue to face coerced contraception, and disabled parents are disproportionately denied child custody. Health system policies also continue to shape disabled people's experiences in mental health and addiction treatment and under what circumstances the state can involuntarily commit and restrain them. In Canada, assisted suicide laws and the ways they might coerce disabled people, especially in the absence of safeguards and input from disabled communities, have also been debated as a form of contemporary eugenics.^39^ Lastly, many disabled people have been forced to live in congregate care settings due to historical and contemporary disinvestment in community-based services (resulting from institutionalization) where they were largely unprotected during the COVID-19 pandemic and died at high rates.^3^

### Potential proximate pathways

#### Health services

Policies and other upstream pathways of structural ableism may contribute to health inequities for and among disabled people via more proximate pathways. Principally, a disabled person's access to health care and whether the services they receive are disability competent and affirming can directly affect their health status.^10^, ^12^ Structural factors such as the physical accessibility of facilities, the accessibility of scheduling platforms and administrative processes, communication access, the training and experience of health professionals around working with disabled patients, and the availability of disabled health professionals to provide disability-congruent health services can all influence quality of care and determine health outcomes for disabled patients.^6^^,^^7^^,^^12^^,^^27^^,^^29^^,^^40^

#### Social determinants and inaccessibility

Another pathway that may connect structural ableism to health is the discrimination and barriers that disabled people encounter in accessing housing, education, employment, and other social determinants of health.^3^ A large body of research has established that these social determinants affect health in multiple ways.^3^^,^^8^^,^^9^ Inaccessibility in the built environment (e.g., in transportation, infrastructure, and public spaces) and in virtual environments (i.e., the web) can also limit access to health services and health-promoting resources for people with physical disabilities, with low vision, who are deaf or hard of hearing, and/or who have other access needs.^40^

#### Adverse childhood experiences and victimization

Throughout society, disabled people are victimized at high rates. Disabled children are nearly twice as likely to have adverse childhood experiences than non-disabled children,^41^ and disabled adults are more than two and a half times more likely to experience victimization, such as rape, sexual assault, and robbery.^42^ While experiences vary by disability type, most communities of disabled people report elevated exposure to violence.^42^

#### State-sanctioned violence

Finally, disabled people, especially Black, Indigenous, and disabled people of color, are unequally exposed to state violence through incarceration, arrest, police brutality, war, and other practices and systems.^3^^,^^16^^,^^22^^,^^42^^,^^43^ In the U.S., the cumulative probability of having been arrested by the age of 28 years is markedly higher among disabled people (average: 42.7%; Black: 55.2%; Hispanic: 46.1%; white: 39.7%) than non-disabled people (average: 29.7%; Black: 37.3%; Hispanic: 31.4%; white: 27.6%).^44^ Furthermore, approximately 66% of incarcerated people in state and federal prisons report a disability, and 42% report a disability and also identify as Black, Hispanic, and/or another minoritized racialized and/or ethnic identity.^43^ While public data on deaths in police custody are incomplete and lack detail on disability status, researchers estimate that more than one-third of persons killed by law enforcement are disabled or have a history of mental illness.^42^

### Potential physiological and behavioral processes

In addition to the pathways discussed above, structural ableism may influence health via complex physiological and behavioral processes, similar to other structural determinants of health.^19^^,^^45^ Some potential processes that may warrant further research include chronic stress and allostatic load, minority stress and the health effects of trauma, and unhealthy substance use and other health risk behaviors as a coping strategy for discrimination experiences and inequitable social conditions.^3^^,^^5^^,^^45^

## Principles for studying structural ableism

**Fig. 2:**
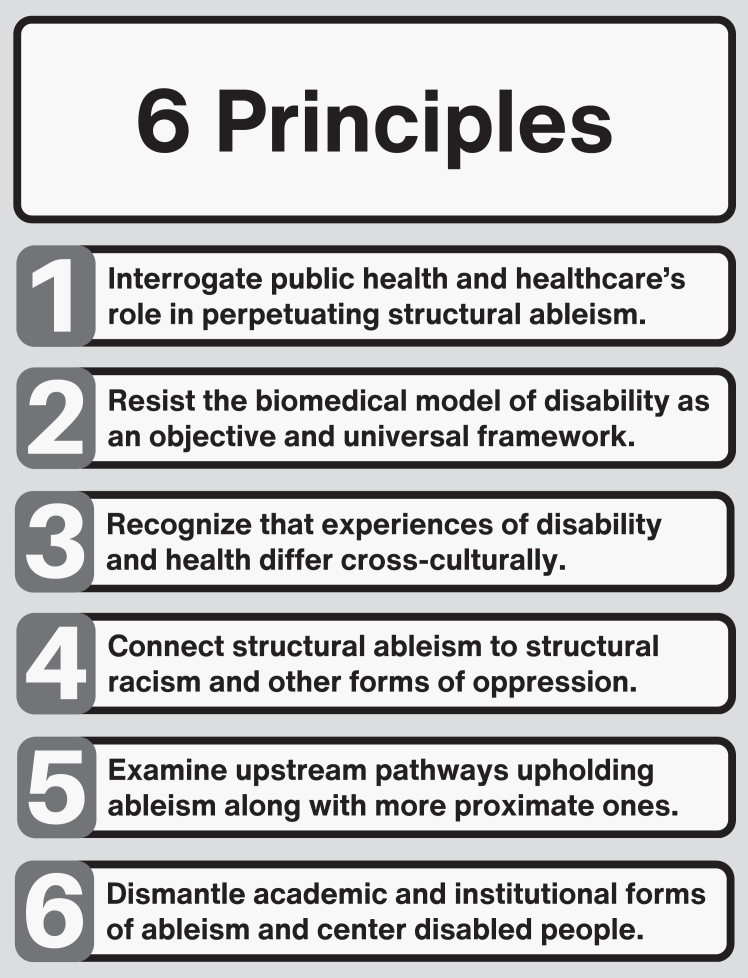
A series of principles relevant to studying structural ableism within health systems.

Despite the growing evidence base around health disparities affecting disabled people, a sizable gap remains around how structural ableism has caused and contributed to these inequities.^1^^,^^2^ Fig. 2 describes a series of principles that hold relevance for studying structural ableism within health systems and its health effects for and among disabled people and other populations impacted by ableism.

### Interrogate public health and healthcare's role in perpetuating structural ableism

While the public health and healthcare fields have often positioned themselves as allies to disabled people, these fields have had a significant and sustained role in perpetuating and legitimizing structural ableism.^1^^,^^2^^,^^6^^,^^36^ While many scientific disciplines and social sectors have been complicit in upholding structural ableism, the public health and healthcare fields are particularly relevant for interrogation given that they provide health services to disabled people and shape public perceptions about what disability is. In the U.S., prior research shows that 32% of health care professionals hold explicit preferences for non-disabled people over disabled people and 84% hold implicit preferences.^6^ Furthermore, less than half of physicians report that they feel very confident providing the same quality of care to disabled patients as non-disabled patients, and most medical school training does not equip students with the tools needed to provide high-quality care to disabled patients.^7^^,^^12^ Thus, before public health and healthcare institutions can effectively contribute to dismantling structural ableism in their fields, it is critical that members of these institutions commit to interrogating the ways that they continue to uphold ableism in their own communities.^29^, ^30^, ^31^, ^32^ This includes listening to the experiences of disabled people within their workforces and responding to their needs and leadership.

### Resist the biomedical model of disability as an objective and universal framework

The biomedical model of disability—which remains engrained in public health and healthcare—is out-of-touch with the work of disability studies scholars who stress the role of social context in shaping a person's experience with disability and ableism.^13^ Nevertheless, many health measures, such as the quality-adjusted life year, the disability-adjusted life year, years of healthy life lost due to disability, and disability free life expectancy continue to privilege the biomedical model in ways that appear likely to devalue disabled people's lives in health policy and generate inaccurate knowledge about disabled people's needs and experiences.^34^ In studying structural ableism, researchers should resist the biomedical model as the only framework for understanding disability and avoid measures that privilege it without sufficient justification.^13^^,^^34^ Instead, we encourage use of the social model of disability as a starting point for interrogating the biomedical model and recommend engagement with disability studies and disability justice scholarship to determine the most relevant models for use, which may differ by context and community.

### Recognize that experiences of disability and health differ cross-culturally

In public health and healthcare, Eurocentric conceptions of disability—chiefly the biomedical model but also the social and human rights models—are often described as universal.^35^, ^36^, ^37^ Scholars such as Maya Kalyanpur and Shridevi Rao have argued that imposing such understandings of disability on Indigenous and/or Global South communities can contribute to erasure of Indigenous knowledge, ignore community strengths, and reinforce colonial patterns of thinking that attempt to homogenize human differences.^35^ Their work highlights the need for improved public health and healthcare approaches that are more attentive to existing local strengths and the historical, cultural, and structural contexts that shape community experiences. In a similar way, researcher and activist Bhargavi Davar has asserted that a global model of mental health is not possible, given the diversity of lived experiences in the Global South, the psychosocial impacts of social and economic inequality resulting from colonialism, and how the evidence for prioritizing pharmacologic interventions in mental healthcare was largely generated in the Global North.^36^ Given this critique, researchers should increasingly work to recognize the heterogeneous ways in which disability is experienced and understood across social and cultural contexts. Such an approach is likely to lead to public health and health care interventions that benefit multiple communities. For example, education researcher Hollie Mackey has proposed an Indigenous leadership paradigm for dismantling ableism that holds relevance for dismantling ableism in many populations.^37^

### Connect structural ableism to structural racism and other forms of oppression

Prior discourse around ableism by disability justice advocates and scholars has emphasized the historical and contemporary interconnectedness of ableism to racism and other forms of oppression.^16^^,^^20^^,^^22^ For example, in the U.S., the prevalence of disability is higher among Black and Indigenous populations than other racialized and ethnic groups and among transgender adults compared to cisgender adults.^9^ In disability studies, DisCrit is a framework proposed by researcher Subini Ancy Annamma that combines concepts from critical race theory and disability studies to analyze race and disability as interdependent constructs using an intersectional framework.^21^ This work is situated within a long tradition of Black feminist and critical race scholarship and activism in which intersectionality has challenged interlocking systems of power (such as racism and ableism) and their effects on the health of people whose health experiences are shaped by the intersection of these systems.^21^ In line with this framework, researchers should consider the ways that structural ableism may converge with structural racism and other forms of oppression to produce inequitable outcomes for and among disabled people.^16^^,^^20^^,^^22^^,^^24^ Supplementary Table S4 presents a selected list of scholarship on intersectional experiences of ableism.

### Examine upstream pathways upholding ableism along with more proximate ones

In a 2023 publication, researchers Rupa Sheth Valdez and Bonnielin Swenor outlined a strategic map for research on structural ableism in health systems.^1^ We reiterate their calls to measure and examine the effects of structural ableism on health and health care experiences using quantitative and qualitative methods across the life course. We emphasize here that structural ableism should be studied at multiple levels, including the upstream policies, institutions, and systems upholding ableism as described in our conceptual framework. To support these efforts, it is critical that public health and health care institutions name ableism and structural ableism as drivers of health inequities for disabled people and direct appropriate resources to researching and interrupting them.^2^

### Dismantle academic and institutional forms of ableism and center disabled people

Finally, it is well-documented that academic and institutional ableism has contributed to significant under-representation of disabled researchers throughout academia, science, and health research.^15^^,^^29^^,^^31^ Since disabled researchers receive only a small fraction of grant funding and have a lower success rate for funding conditional on applying,^28^ it is plausible that research on structural ableism in public health and healthcare could be dominated by non-disabled researchers. For this reason, it is critical that researchers studying structural ableism acknowledge their positionality, partner with disabled scholars and community members in ways that meaningfully distribute resources and power, read and cite disability studies and disability justice scholarship, and work to disseminate research in ways that are accessible to disabled and deaf communities.^15^^,^^31^^,^^32^

## Concluding remarks

This viewpoint examines scholarship by disability studies and disability justice scholars and researchers studying social and structural determinants of health to offer a definition of structural ableism in public health and healthcare. It also proposes potential pathways through which structural ableism may influence health and several principles for studying structural ableism in health systems. The larger purpose of this viewpoint, however, is to call for increased engagement around ableism as a structural determinant of health to advance more equitable health policies and health care practices for disabled people and other populations impacted by ableism. Ultimately, the most promising strategy to interrupt ableism is to center a representative coalition of disabled people in the solutions.^31^, ^32^

## Contributors

DJL's role included conceptualization, formal analysis, visualization, and writing (original draft, review, and editing). JAC's role included supervision of the study and writing (original draft, review, and editing).

## Declaration of interests

The authors report that they have no conflicts of interests to disclose.

## Appendix A Supplementary data

lundberg_ableism_lancet_RR3_supplemen
