# Supplementary material for: Structural ableism in public health and healthcare: a definition and conceptual framework
Source: Lancet Reg Health Am. 2023 Dec 18;30:100650. doi: 10.1016/j.lana.2023.100650 (PMC10770745; doi:10.1016/j.lana.2023.100650)
Supplement: lundberg_ableism_lancet_RR3_supplemen [file mmc1.docx]

# Supplementary Materials

## Article Information:

Title: “Structural ableism in public health and healthcare: a definition and conceptual framework”

Authors: Dielle J. Lundberg and Jessica A. Chen

Email for Corresponding Author: dielle@uw.edu

## Table of Contents:

Supplementary Text

Section A. Discussion of Language Choices Around Disability

Section B. Approach to Narrative Review

Supplementary Table 1. Frequently Cited Definitions of Structural Ableism

Supplementary Table 2. Selected Literature on Policies Related to Structural Ableism

Supplementary Table 3. Selected Literature on Eugenics, Institutionalization and Health

Supplementary Table 4. Selected Literature on Intersectionality and Ableism

Supplementary References

## Supplementary Text

### Section A. Discussion of Language Choices Around Disability

#### On Diversity and Self-Determination of Language:

Language and self-identification are powerful tools by which marginalized people, including disabled people, come to understand themselves and convey information about their identities, experiences, and communities.^1^ When it comes to identifying the correct language to use for people living with disability, neurodivergence, mental illness, chronic illness, and other related experiences, we suggest considering several points:

First, there is currently no single shared view among disabled people regarding a universal set of terms to describe disability.^1–5^ The disabled community is incredibly diverse, and one of the ways this is reflected is in the many ways that individuals and communities describe their experiences.^6^

Second, discussions surrounding the correct language for disability and related experiences should “centre first and foremost on the needs, autonomy, and rights” of people with these experiences “so in to preserve their rights to self-determination.”^2^ In this way, health professionals, researchers, and policy-makers should respect and use disabled individuals and communities’ chosen language whenever possible and respect how their language choices may evolve over time.^5^ Where an individual or community’s chosen language is not apparent, we encourage researchers to offer a clear discussion around their language choices and if needed to invite feedback.^6^

Third, non-disabled people should avoid critiquing how disabled people choose to self-identify and describe their own experiences.

Fourth, although there is a lack of consensus on a single universal set of terms to describe disability, there are many harmful terms for disability that health professionals, researchers, and policy-makers should avoid. The exception to this are terms which some disabled people are in the process of reclaiming (e.g., mad and crip).^6–9^ Health professionals, researchers, and policy-makers might consider using these terms when they are referring to a disabled person or community who has requested for use of this language, if they are referring to scholarship that uses these terms, and/or if they are referring to their own experiences or to those of a community they identify with.

#### On Identify-First vs. Person-First Language:

Unless a community has requested use of specific language, it is generally acceptable to use identify-first or person-first language when discussing disability at the population-level, as disabled people have differing views on this issue.^5^ Person-first language (i.e., person with a disability) emphasizes the person and highlights disability as an experience that can be independent from a person, which for some disabled people has a destigmatizing role.^3^ Disability-first language (i.e., disabled person) emphasizes disabled identity and highlights how disability can be a social, cultural, and political identity.^1,10^ Additionally, many disabled people do not feel that their disabilities can be separated from who they are.^11^ As autistic activist Jim Sinclair wrote in 1999: “I am usually a ‘person with a purple shirt,’ but I could also be a ‘person with a blue shirt’ one day, and a ‘person with a yellow shirt’ the next day, and I would still be the same person, because my clothing is not part of me. But autism is part of me. Autism is hard-wired into the ways my brain works. I am autistic because I cannot be separated from how my brain works.’”^12^

#### On the Language Choices in Our Viewpoint:

We used our chosen language in this viewpoint. Wherever possible, we also opted for language that is currently used within the public health and health services fields, as health system actors in these fields are the primary intended audience for this article. We used the terms “mad and/or living with mental illness” in an attempt to highlight the diverse ways in which individuals who are mad, psychiatrized, and/or are living with mental illness, mental health conditions, mental disability and/or psychiatric disability conceptualize these experiences.^9^ We wish to directly acknowledge that our language choices in this article do not reflect the language choices of all disabled people.

### Section B. Approach to Narrative Review

#### Objective:

The objective of our narrative review was to identify scholarship that included the term “structural ableism” and a list of authors and publications that were frequently cited in regards to informing definitions and/or discussions of structural ableism. Reviewing all existing literature on social and health inequities for disabled people that provide examples of factors that could be inferred as structural or systemic (e.g., related to policies, institutions, and/or systems) is a larger project that exceeded the scope of this article. In this article, we sought to conduct a narrative review to ensure that we presented a definition of structural ableism tailored to the context of public health and healthcare that captured diverse perspectives and also credited many of the authors whose work has been foundational to current understandings of ableism.

#### Search Strategy and Selection Criteria:

Our search strategy included PubMed and Google Scholar databases for publications that included the term “structural ableism”. We used Google Scholar because it captures the disability studies and education literature where publication about ableism has been more frequent and because it includes non-peer-reviewed publications. Many disabled scholars and activists have faced barriers in academic publishing and thus have disseminated their work in channels outside of peer-reviewed academic journals.^13^ Our search occurred in February 2023. Our search yielded 242 publications that included the term “structural ableism,” of which we were able to access 209 full texts that were written in English or included an English translation to review and determine if they defined, conceptualized, or measured structural dimensions of ableism. We identified 49 publications which defined, conceptualized, or measured structural dimensions of ableism, which we analyzed further. We then extracted 5 types of data from the included publications: (a) definitions of structural ableism, (b) definitions of ableism that discussed institutions, structures, and/or systems, (c) the names and authors of studies that were cited as informants or original sources for these definitions, (d) text related to how ableism connects to other concepts, and (e) text related to how ableism and/or structural ableism can be measured.

#### Synthesis into a Definition:

Among the authors and studies frequently named as informants or original sources of knowledge around structural ableism in the included articles, we selected 10 that were cited frequently. In cases where an author had multiple relevant cited publications, we selected the publication that in our view had the most in-depth definition of structural ableism (or discussion of ableism functioning structurally via policies, institutions, and/or systems). **Supplemental Table 1** captures relevant text about the definition and/or discussion of structural ableism from each of these publications. In synthesizing concepts from these definitions of structural ableism into a definition of structural ableism tailored for public health and healthcare, we chose to prioritize more recent definitions because these definitions often referenced older definitions and added to them. We also chose to prioritize definitions that discussed intersectionality because we sought to offer a definition that is relevant to multiple communities of disabled people, including those who live at the intersection of multiple systems of oppression.

#### Limitations:

Structural ableism can be defined in nuanced ways depending on the discipline, context, and community, and the definition we offer in this viewpoint will not be suitable for all cases. For this reason, definitions should be further refined over time and adapted for various disciplines, contexts, and communities of disabled people and other populations impacted by ableism. Structural audism and structural sanism are examples of related and/or more narrow terms, for which tailored definitions are useful.^9,14^ Future research should explore how structural ableism operates differently to influence health and health systems in each of these disciplines, contexts, and communities. When citing our definition of structural ableism, we encourage scholars to also cite the disability studies and disability justice scholars that we name and reference. A central goal of our article is to encourage health system actors to engage with disability studies and disability justice scholarship, including literature we cite here and in our main article.

## Supplementary Table 1. Frequently Cited Definitions of Structural Ableism

### This table is presented in list form for increased accessibility.

1. Annamma (2012)^15^
   1. “We believe, for instance, that racism and ableism are normalizing processes that are interconnected and collusive. In other words, racism and ableism often work in ways that are unspoken, yet racism validates and reinforces ableism, and ableism validates and reinforces racism. For students of color, race does not exist outside of ability and ability does not exist outside of race; each is being built upon the perception of the other. However, because racism and ableism are ‘so enmeshed in the fabric of our social order, [they] appear both normal and natural to people in this culture’... A DisCrit theory in education is a framework that theorizes about the ways in which race, racism, dis/ability and ableism are built into the interactions, procedures, discourses, and institutions of education, which affect students of color with dis/abilities qualitatively differently than white students with dis/abilities.”
2. Berne (Sins Invalid) (2018)^16^
   1. “We know that each person has multiple identities, and that each identity can be a site of privilege or oppression. The mechanical workings of oppression and how they output shift depending upon the characteristics of any given institutional or interpersonal interaction; the very experience of disability itself is being shaped by race, gender, class, gender expression, historical moment, relationship to colonization, and more… We know ableism exists in the context of other historical systemic oppressions. We know to truly have liberation we must be led by those who know the most about these systems and how they work.”
3. Bogart (2019)^17^
   1. “We define ableism as stereotyping, prejudice, discrimination, and social oppression toward people with disabilities—the largest minority group in the United States. People with disabilities are broadly defined as those who have conditions that are commonly perceived to be disabilities by the general public, including physical, sensory, and intellectual disabilities, in addition to invisible disabilities, chronic health conditions, psychiatric conditions, and others.”
4. Brown (2020)^18^
   1. “Ableism is discrimination in favour of able-bodied people, people who are not ill, who do not have a disability, who are neurotypical. Ableism is the discrimination and social prejudice against people who fall outside those normal boundaries. Ableism characterises such people as defined by their disabilities and sees them as inferior; unfortunately, it is endemic within the culture and fabric of academia. It is up to all of us to challenge this, to acknowledge our human complexities and fluctuations, including the invisible parts of the iceberg of experience, to celebrate the vivid diversity already present in the academy, while striving for more, and to help make change.”
5. Campbell (2001)^19^
   1. “A network of beliefs, processes and practices that produce a particular kind of self and body (the corporeal standard) that is projected as the perfect, species-typical and therefore essential and fully human. Disability, then, is cast as a diminished state of being human.”
6. Chouinard (1997)^20^
   1. “Ableism refers to ideas, practices, institutions, and social relations that presume able-bodiedness, and by so doing, construct persons with disabilities as marginalized, oppressed, and largely invisible 'others'. This presumption, whether intentional or not, means that one's ability to approximate the able-bodied norm, influences multiple facets of life: such as the character and quality of interpersonal relations, economic prospects, and degrees of physical and social access to various life spaces. Ableism entails a way of being that takes mobility, thinking, speech, and the senses for granted, and which includes largely 'unconscious' aversion to people and bodies that remind us that the able-bodied norm is an ideal... that we are all mortal and subject to disease and death... An ableist society is, then, one that tends to devalue its non-able-bodied members; despite good intentions on the part of many of its citizens to treat these 'others' as equals.”
7. Friedman (2017)^21^
   1. “Ableism [is] the compulsory preference for non-disability… Ableism, like other "isms" such as racism and sexism, describes discrimination towards a social group, in this case disabled people, but it also describes how certain ideals and attributes are valued or not valued... For example, walking is more socially valued than moving by way of wheelchair... Disabled people have expressed that the negative effects of ableism have been socially and psychologically damaging to them in a number of ways. What is less known, however, is how ableist attitudes and understandings of disability appear in other groups that may not identify as disabled.”
8. Goodley (2014)^22^
   1. “Ableism… accounts for the stifling practices associated with a contemporary society that increasingly seeks to promote…the ‘species-typical individual citizen’: a citizen that is ready and able to work and contribute; an atomistic phenomenon cut off from others, capable, malleable and compliant. Ableism breeds paranoia, confusion, fear and inadequacy. Ableism is an ideal that no one ever matches up to… Compulsory ableism is to disablism what compulsory heterosexuality is to homophobia. Ableism provides just the right amount of temperature and nutrients for disablism to grow. As we study ableism, this engenders an analytical turn away from disability to ask: what do we mean by being able? What is valued by being as able as possibly or ideally one could be? Ableism is equally an individual and a global project.”
9. Lewis (2022)^23^
   1. “A system of assigning value to people's bodies and minds based on societally constructed ideas of normalcy, productivity, desirability, intelligence, excellence, and fitness. These constructed ideas are deeply rooted in eugenics, anti-Blackness, misogyny, colonialism, imperialism, and capitalism. This systemic oppression that leads to people and society determining people's value based on their culture, age, language, appearance, religion, birth or living place, ‘health/wellness’, and/or their ability to satisfactorily re/produce, ‘excel’ and ‘behave.’ You do not have to be disabled to experience ableism.”
10. Mingus (2011)^10^
    1. “Ableism must be included in our analysis of oppression and in our conversations about violence, responses to violence and ending violence. Ableism cuts across all of our movements because ableism dictates how bodies should function against a mythical norm—an able-bodied standard of white supremacy, heterosexism, sexism, economic exploitation, moral/religious beliefs, age and ability. Ableism set the stage for queer and trans people to be institutionalized as mentally disabled; for communities of color to be understood as less capable, smart and intelligent, therefore “naturally” fit for slave labor; for women’s bodies to be used to produce children, when, where and how men needed them; for people with disabilities to be seen as “disposable” in a capitalist and exploitative culture because we are not seen as “productive;” for immigrants to be thought of as a “disease” that we must “cure” because it is “weakening” our country; for violence, cycles of poverty, lack of resources and war to be used as systematic tools to construct disability in communities and entire countries.”

Footnote: This list is not comprehensive and is intended to provide a selected list of examples of relevant prior scholarship.

## Supplementary Table 2. Selected Literature on Policies Related to Structural Ableism

### This table is presented in list form for increased accessibility.

1. Economic Policies
   1. Worth Less? Exploring the Effects of Subminimum Wages on Poverty among U.S. Hourly Workers^24^
   2. The Impacts of Paid Family and Medical Leave on Worker Health, Family Well-Being, and Employer Outcomes^25^
   3. Poverty among adults with disabilities: barriers to promoting asset accumulation in individual development accounts^26^
   4. Dismantling the Poverty Trap: Disability Policy for the Twenty-First Century^27^
   5. How Dehumanizing Administrative Burdens Harm Disabled People^28^
   6. America’s housing affordability crisis: Perpetuating disparities among people with disability^29^
   7. Disability Inclusive Employment and the Accommodation Principle: Emerging Issues in Research, Policy, and Law^30^
   8. Cripping the "Crack Baby" Epidemic: A Feminist Disability Genealogy of Welfare Reform^31^
2. Social Policies
   1. Rights Not Fundamental: Disability and the Right to Marry^32^
   2. Assessment of Individuals with Disabilities in Latin America: a Comparative Study of the Legislation^33^
   3. Let's Try Again: Why the United States Should Ratify the United Nations Convention on the Rights of People with Disabilities^34^
   4. How people with disabilities experience programs to prevent intimate partner violence across four countries^35^
3. Health Policies
   1. U.S. alcohol treatment admissions after the Mental Health Parity and Addiction Equity Act: Do state parity laws and race/ethnicity make a difference?^36^
   2. State mental health insurance parity laws and college educational outcomes^37^
   3. Medicaid Expansion And State Trends In Supplemental Security Income Program Participation^38^
   4. Medical Assistance in Dying: A Review of Related Canadian News Media Texts^39^
   5. Out-of-Pocket Health Expenditures Associated with Chronic Health Conditions and Disability in China^40^
   6. The Variations in Catastrophic and Impoverishing Health Expenditures, and Its Determinants in Iran: A Scoping Review^41^
   7. Disability inclusiveness of government responses to COVID-19 in South America: a framework analysis study^42^
   8. US Physicians’ Knowledge About The Americans With Disabilities Act And Accommodation Of Patients With Disability^43^
   9. Triple jeopardy: disabled people and the COVID-19 pandemic^44^
   10. Radical Health: Unwellness, Care, and Latinx Expressive Culture^45^
   11. Equitable Access to Telehealth and Other Services for Deaf People During the COVID-19 Pandemic^46^
4. Educational Policies
   1. The impact of inclusive education reforms on students with disability: an international comparison^47^
   2. From Detractive to Democratic: The Duty of Teacher Education to Disrupt Structural Ableism and Reimagine Disability^48^
   3. Addressing ableism in inclusive education policies: a policy brief outlining Italy, Poland, the Netherlands and the United Kingdom^49^
   4. Avoiding Linguistic Neglect of Deaf Children^50^
   5. Discourses of prejudice in the professions: the case of sign languages^51^
5. Built and Virtual Environment Policy
   1. Web Accessibility for Older Adults: A Comparative Analysis of Disability Laws^52^
   2. Accessible at last?: what do new European digital accessibility laws mean for disabled people in the UK?^53^
   3. Investigating accessibility in Latin American science museums and centers^54^
   4. Transportation challenges for persons aging with mobility disability: Qualitative insights and policy implications^55^
   5. Are communities in the United States planning for pedestrians with disabilities? Findings from a systematic evaluation of local government barrier removal plans^56^

Footnote: This list is not comprehensive and is intended to provide a selected list of examples of relevant prior scholarship. The categories used to classify the policies in this table are intended to direct the reader to areas of interest; however, many of these policies cut across multiple sectors.

## Supplementary Table 3. Selected Literature on Eugenics, Institutionalization and Health

### This table is presented in list form for increased accessibility.

1. Eugenics Informing Health Policy
   1. Confronting Eugenics Means Finally Confronting Its Ableist Roots^57^
   2. The devolution of eugenic practices: Sexual and reproductive health and oppression of people with intellectual disability^58^
   3. The Public and Private History of Eugenics: An Introduction^59^
   4. Eugenics and its Relevance to Contemporary Health Care^60^
   5. The Legacy of Eugenics in Contemporary Law^61^
   6. From preventive eugenics to slippery eugenics: Population control and contemporary sterilisations targeted to indigenous peoples in Mexico^62^
   7. Reproductive injustice, trans rights, and eugenics^63^
   8. Child Protective Service Disparities and Serious Mental Illnesses: Results From a National Survey^64^
   9. Deviancy, Dependency, and Disability: the Forgotten History of Eugenics and Mass Incarceration^65^
2. Institutionalization Informing Health Policy
   1. Understanding psychiatric institutionalization: a conceptual review^66^
   2. Understanding the History of Institutionalization: Making connections to De-institutionalization and the Olmstead Act for Persons with Intellectual Disabilities in the State of Illinois^67^
   3. “Places of Such Towering Misery”: The History of the Institutionalization of Disabled People and Deinstitutionalization^68^
   4. Disability and Native North American Boarding School Narratives: Madonna Swan and Sioux Sanitorium^69^

Footnote: This list is not comprehensive and is intended to provide a selected list of examples of relevant prior scholarship.

## Supplementary Table 4. Selected Literature on Intersectionality and Ableism

### This table is presented in list form for increased accessibility.

1. Some Foundational Texts on Intersectionality
   1. Demarginalizing the Intersection of Race and Sex: A Black Feminist Critique of Antidiscrimination Doctrine, Feminist Theory and Antiracist Politics^70^
   2. Black feminist thought: Knowledge, consciousness, and the politics of empowerment^71^
   3. The Combahee River Collective statement^72^
   4. The problem with the phrase women and minorities: intersectionality-an important theoretical framework for public health^73^
   5. Dis/ability critical race studies (DisCrit): theorizing at the intersections of race and dis/ability^15^
   6. Ten Principles of Disability Justice^16^
   7. Skin, Tooth, and Bone: The Basis of Movement is Our People: a Disability Justice Primer^74^
   8. Moving Toward the Ugly: A Politic Beyond Desirability^10^
   9. Crip Genealogies^75^
   10. On Fits, Starts, and Entry Points: The Rise of Black Disability Studies^76^
   11. Integrating Race, Transforming Feminist Disability Studies^77^
2. Intersectional Experiences of Ableism
   1. Discrimination at every turn: An intersectional ecological lens for rehabilitation^78^
   2. We still cannot breathe: Applying intersectional ecological model to COVID-19 survivorship^79^
   3. Tools of Exclusion: Race, Disability, and (Re)segregated Education^80^
   4. Disability as a Colonial Construct: The Missing Discourse of Culture in Conceptualizations of Disabled Indigenous Children^81^
   5. Disability and other identities?-how do they intersect?^82^
   6. Intersectionality Within Critical Autism Studies: A Narrative Review^83^
   7. “Tuned Into a Different Channel”: Autistic Transgender Adults’ Experiences of Intersectional Stigma^84^
   8. Health Care Disparities Among Autistic LGBTQ+ People^85^
   9. Indigenous Perspectives on Disability^86^
   10. Crip Theory: Cultural Signs of Queerness and Disability^7^
   11. Health Disparities Among Mid-to-Older Deaf LGBTQ Adults Compared with Mid-to-Older Deaf Non-LGBTQ Adults in the United States^87^
   12. The next critical turn for ageism research: The intersections of ageism and ableism^88^
   13. "Under great anxiety": Pregnancy experiences of Vietnamese women with physical disabilities seen through an intersectional lens^89^
   14. #BodyPositive? A critical exploration of the body positive movement within physical cultures taking an intersectionality approach^90^
   15. The Intersectionality of Disability, Religion, Ethnicity, and Gender: Muslim Arabs in the United States^91^
   16. Disability and Ethnicity: how young Asian disabled people make sense of their lives^92^
   17. The Intersectionality of Ethnicity/race and Intellectual and Developmental Disabilities: Impact on Health Profiles, Service Access and Mortality^93^
   18. Disability and Forced Migration: Critical Intersectionalities^94^
   19. Race, Ethnicity and Disability: Charting Complex and Intersectional Terrains^95^

Footnote: This list is not comprehensive and is intended to provide a selected list of examples of relevant prior scholarship.

## Supplementary References

1. Grech LB, Koller D, Olley A. Furthering the person-first versus identity-first language debate. Aust Psychol [Internet]. 2023 Jul 4;58(4):223–32. Available from: <https://doi.org/10.1080/00050067.2023.2192863>

2. Botha M, Hanlon J, Williams GL. Does Language Matter? Identity-First Versus Person-First Language Use in Autism Research: A Response to Vivanti. J Autism Dev Disord [Internet]. 2023 Feb;53(2):870–8. Available from: <http://dx.doi.org/10.1007/s10803-020-04858-w>

3. Dunn DS, Andrews EE. Person-first and identity-first language: Developing psychologists’ cultural competence using disability language. Am Psychol [Internet]. 2015 Apr;70(3):255–64. Available from: <http://dx.doi.org/10.1037/a0038636>

4. Duncan J, O’Neill R. Person-first, identity-first and the language of deafness. Deafness & Education International [Internet]. 2020 Jan 2;22(1):1–2. Available from: <https://doi.org/10.1080/14643154.2020.1720204>

5. Writing Respectfully: Person-First and Identity-First Language [Internet]. National Institutes of Health (NIH). 2023 [cited 2023 Sep 29]. Available from: <https://www.nih.gov/about-nih/what-we-do/science-health-public-trust/perspectives/writing-respectfully-person-first-identity-first-language>

6. Best KL, Mortenson WB, Lauzière-Fitzgerald Z, Smith EM. Language matters! The long-standing debate between identity-first language and person first language. Assist Technol [Internet]. 2022 Mar 4;34(2):127–8. Available from: <http://dx.doi.org/10.1080/10400435.2022.2058315>

7. McRuer R. Crip theory: Cultural signs of queerness and disability. NYU press; 2006.

8. McWade B, Milton D, Beresford P. Mad studies and neurodiversity: a dialogue. Disabil Soc [Internet]. 2015 Feb 7;30(2):305–9. Available from: <https://doi.org/10.1080/09687599.2014.1000512>

9. Nabbali EM. A “Mad” Critique of the Social Model of Disability. International Journal of Diversity in Organizations, Communities, and Nations; Patras [Internet]. 2009;9(4):1–12. Available from: <http://dx.doi.org/10.18848/1447-9532/CGP/v09i04/39702>

10. Mingus M. Moving Toward the Ugly: A Politic Beyond Desirability [Internet]. Leaving Evidence. 2011 [cited 2023 Feb 18]. Available from: <https://leavingevidence.wordpress.com/2011/08/22/moving-toward-the-ugly-a-politic-beyond-desirability/>

11. Liebowitz C. I am Disabled: On Identity-First Versus People-First Language [Internet]. The Body Is Not An Apology. 2015 [cited 2023 Sep 28]. Available from: <https://thebodyisnotanapology.com/magazine/i-am-disabled-on-identity-first-versus-people-first-language/>

12. Sinclair J. Why I dislike “person first” language [Internet]. Autism Mythbusters. 1999 [cited 2023 Sep 28]. Available from: <https://autismmythbusters.com/general-public/autistic-vs-people-with-autism/jim-sinclair-why-i-dislike-person-first-language/>

13. Brown N, Leigh J. Ableism in academia: where are the disabled and ill academics? Disabil Soc [Internet]. 2018 Jul 3;33(6):985–9. Available from: <https://doi.org/10.1080/09687599.2018.1455627>

14. Perrodin-Njoku E, Corbett C, Moges-Riedel R, Simms L, Kushalnagar P. Health disparities among Black deaf and hard of hearing Americans as compared to Black hearing Americans: A descriptive cross-sectional study. Medicine [Internet]. 2022 Jan 14;101(2):e28464. Available from: <http://dx.doi.org/10.1097/MD.0000000000028464>

15. Annamma SA, Connor D, Ferri B. Dis/ability critical race studies (DisCrit): theorizing at the intersections of race and dis/ability. Race Ethnicity and Education [Internet]. 2012;16(1):1–31. Available from: <https://doi.org/10.1080/13613324.2012.730511>

16. Berne P, Morales AL, Langstaff D, Sins Invalid. Ten Principles of Disability Justice. WSQ: Women’s Studies Quarterly [Internet]. 2018;46(1):227–30. Available from: <http://dx.doi.org/10.1353/wsq.2018.0003>

17. Bogart KR, Dunn DS. Ableism Special Issue Introduction. J Soc Issues [Internet]. 2019 Sep 1;75(3):650–64. Available from: <https://doi.org/10.1111/josi.12354>

18. Brown N, Leigh J, editors. Ableism in Academia: Theorising experiences of disabilities and chronic illnesses in higher education [Internet]. UCL Press; 2020. Available from: <https://doi.org/10.2307/j.ctv13xprjr>

19. Campbell FK. Inciting Legal Fictions: “Disability”s’ date with Ontology and the Ableist Body of Law. Griffith Law Review [Internet]. 2001 [cited 2023 Sep 29];10(1). Available from: <http://hdl.handle.net/10072/3714>

20. Chouinard V. Making Space for Disabling Differences: Challenging Ableist Geographies: Introduction: Situating Disabling Differences. Environ Plan D [Internet]. 1997 Aug 1;15(4):379–87. Available from: <https://doi.org/10.1068/d150379>

21. Friedman C, Owen AL. Defining Disability: Understandings of and Attitudes Towards Ableism and Disability. Disabil Stud Q [Internet]. 2017 Mar 7 [cited 2023 Mar 17];37(1). Available from: <https://doi.org/10.18061/dsq.v37i1.5061>

22. Goodley D. Dis/ability studies: Theorising disablism and ableism. Routledge; 2014.

23. Lewis TA. Working Definition of Ableism - January 2022 Update [Internet]. Talila A. Lewis. 2022 [cited 2023 Nov 22]. Available from: <https://www.talilalewis.com/blog/working-definition-of-ableism-january-2022-update>

24. Maroto M, Pettinicchio D. Worth Less? Exploring the Effects of Subminimum Wages on Poverty among U.S. Hourly Workers. Sociol Perspect [Internet]. 2023 Jun 1;66(3):455–75. Available from: <https://doi.org/10.1177/07311214221124630>

25. Bartel A, Rossin-Slater M, Ruhm C, Slopen M, Waldfogel J. The Impacts of Paid Family and Medical Leave on Worker Health, Family Well-Being, and Employer Outcomes. Annu Rev Public Health [Internet]. 2023 Apr 3;44:429–43. Available from: <http://dx.doi.org/10.1146/annurev-publhealth-071521-025257>

26. Soffer M, McDonald KE, Blanck P. Poverty among adults with disabilities: barriers to promoting asset accumulation in individual development accounts. Am J Community Psychol [Internet]. 2010 Dec;46(3-4):376–85. Available from: <http://dx.doi.org/10.1007/s10464-010-9355-4>

27. Stapleton DC, O’Day BL, Livermore GA, Imparato AJ. Dismantling the poverty trap: disability policy for the twenty-first century. Milbank Q [Internet]. 2006;84(4):701–32. Available from: <http://dx.doi.org/10.1111/j.1468-0009.2006.00465.x>

28. Bonitatibus S, DiMatteo E, Buffie N, Ives-Rublee M. How Dehumanizing Administrative Burdens Harm Disabled People [Internet]. Center for American Progress. 2022 [cited 2023 Sep 28]. Available from: <https://www.americanprogress.org/article/how-dehumanizing-administrative-burdens-harm-disabled-people/>

29. Burns SP, Mendonca R, Pickens ND, Smith RO. America’s housing affordability crisis: Perpetuating disparities among people with disability. Disabil Soc [Internet]. 2021 Aug;36. Available from: <http://dx.doi.org/10.1080/09687599.2021.1960276>

30. Blanck P. Disability Inclusive Employment and the Accommodation Principle: Emerging Issues in Research, Policy, and Law. J Occup Rehabil [Internet]. 2020 Dec;30(4):505–10. Available from: <http://dx.doi.org/10.1007/s10926-020-09940-9>

31. Frye L. Cripping the “Crack Baby” Epidemic: A Feminist Disability Genealogy of Welfare Reform. ff [Internet]. 2022 [cited 2023 Oct 4];34(2):69–98. Available from: <http://dx.doi.org/10.1353/ff.2022.0023>

32. Garbero G. Rights Not Fundamental: Disability and the Right to Marry. Louis UJ Health L & Pol’y [Internet]. 2020;14:587. Available from: <https://scholarship.law.slu.edu/jhlp/vol14/iss2/17>

33. Rocha RN das, Fernandes FC, Nunes R. Assessment of Individuals with Disabilities in Latin America: a Comparative Study of the Legislation. International Archives of Medicine Section: Medical Humanities [Internet]. 2020; Available from: <http://dx.doi.org/10.3823/2630>

34. Kanter AS. Let’s Try Again: Why the United States Should Ratify the United Nations Convention on the Rights of People with Disabilities. Touro Law Rev [Internet]. 2019 [cited 2023 Sep 29];35. Available from: <https://papers.ssrn.com/sol3/papers.cfm?abstract_id=3373259>

35. Stern E, van der Heijden I, Dunkle K. How people with disabilities experience programs to prevent intimate partner violence across four countries. Eval Program Plann [Internet]. 2020 Apr;79:101770. Available from: <http://dx.doi.org/10.1016/j.evalprogplan.2019.101770>

36. Mulia N, Lui CK, Ye Y, Subbaraman MS, Kerr WC, Greenfield TK. U.S. alcohol treatment admissions after the Mental Health Parity and Addiction Equity Act: Do state parity laws and race/ethnicity make a difference? J Subst Abuse Treat [Internet]. 2019 Nov;106:113–21. Available from: <http://dx.doi.org/10.1016/j.jsat.2019.08.008>

37. Solomon KT, Dasgupta K. State mental health insurance parity laws and college educational outcomes. J Health Econ [Internet]. 2022 Dec;86:102675. Available from: <http://dx.doi.org/10.1016/j.jhealeco.2022.102675>

38. Soni A, Burns ME, Dague L, Simon KI. Medicaid Expansion And State Trends In Supplemental Security Income Program Participation. Health Aff [Internet]. 2017 Aug 1;36(8):1485–8. Available from: <http://dx.doi.org/10.1377/hlthaff.2016.1632>

39. Brassolotto J, Manduca-Barone A, Zurbrigg P. Medical Assistance in Dying: A Review of Related Canadian News Media Texts. J Med Humanit [Internet]. 2023 Jun;44(2):167–86. Available from: <http://dx.doi.org/10.1007/s10912-022-09764-z>

40. Gao J, Kim H, Mitra S. Out-of-Pocket Health Expenditures Associated with Chronic Health Conditions and Disability in China. Int J Environ Res Public Health [Internet]. 2023 Jul 27;20(15). Available from: <http://dx.doi.org/10.3390/ijerph20156465>

41. Hedayati M, Masoudi Asl I, Maleki M, Fazaeli AA, Goharinezhad S. The Variations in Catastrophic and Impoverishing Health Expenditures, and Its Determinants in Iran: A Scoping Review. Med J Islam Repub Iran [Internet]. 2023 Apr 26;37:44. Available from: <http://dx.doi.org/10.47176/mjiri.37.44>

42. Sakellariou D, Malfitano APS, Rotarou ES. Disability inclusiveness of government responses to COVID-19 in South America: a framework analysis study. Int J Equity Health [Internet]. 2020 Aug 3;19(1):131. Available from: <http://dx.doi.org/10.1186/s12939-020-01244-x>

43. Iezzoni LI, Rao SR, Ressalam J, Bolcic-Jankovic D, Agaronnik ND, Lagu T, et al. US Physicians’ Knowledge About The Americans With Disabilities Act And Accommodation Of Patients With Disability. Health Aff [Internet]. 2022 Jan;41(1):96–104. Available from: <http://dx.doi.org/10.1377/hlthaff.2021.01136>

44. Shakespeare T, Ndagire F, Seketi QE. Triple jeopardy: disabled people and the COVID-19 pandemic. Lancet [Internet]. 2021 Apr 10;397(10282):1331–3. Available from: <http://dx.doi.org/10.1016/S0140-6736(21)00625-5>

45. Minich JA. Radical Health: Unwellness, Care, and Latinx Expressive Culture. Duke University Press; 2023. 232 p.

46. Moreland CJ, Rao SR, Jacobs K, Kushalnagar P. Equitable Access to Telehealth and Other Services for Deaf People During the COVID-19 Pandemic. Health Equity [Internet]. 2023 Feb 28;7(1):126–36. Available from: <http://dx.doi.org/10.1089/heq.2022.0115>

47. de Bruin K. The impact of inclusive education reforms on students with disability: an international comparison. International Journal of Inclusive Education [Internet]. 2019 Aug 3;23(7-8):811–26. Available from: <https://doi.org/10.1080/13603116.2019.1623327>

48. Keefe ES. From Detractive to Democratic: The Duty of Teacher Education to Disrupt Structural Ableism and Reimagine Disability. Teach Coll Rec [Internet]. 2022 Mar 1;124(3):115–47. Available from: <https://doi.org/10.1177/01614681221086994>

49. Kusters J, Millner MA, Omelyanovskaya K, Tangerli MM, Laszewska A, van Kessel R. Addressing ableism in inclusive education policies: a policy brief outlining Italy, Poland, the Netherlands and the United Kingdom. SEEJPH [Internet]. 2021 Aug 19 [cited 2023 Sep 29]; Available from: <https://doi.org/10.11576/seejph-4681>

50. Humphries T, Kushalnagar P, Mathur G, Napoli DJ, Padden C, Rathmann C, et al. Avoiding Linguistic Neglect of Deaf Children. Soc Serv Rev [Internet]. 2016 Dec 1;90(4):589–619. Available from: <https://doi.org/10.1086/689543>

51. Kushalnagar P, Topolski TD, Schick B, Edwards TC, Skalicky AM, Patrick DL. Mode of communication, perceived level of understanding, and perceived quality of life in youth who are deaf or hard of hearing. J Deaf Stud Deaf Educ [Internet]. 2011 May 2;16(4):512–23. Available from: <http://dx.doi.org/10.1093/deafed/enr015>

52. Yang YT, Chen B. Web Accessibility for Older Adults: A Comparative Analysis of Disability Laws. Gerontologist [Internet]. 2015 Oct;55(5):854–64. Available from: <http://dx.doi.org/10.1093/geront/gnv057>

53. Lewthwaite S, James A. Accessible at last?: what do new European digital accessibility laws mean for disabled people in the UK? Disabil Soc [Internet]. 2020 Sep 13;35(8):1360–5. Available from: <https://doi.org/10.1080/09687599.2020.1717446>

54. Rocha JN, Massarani L, Abreu WVDE, Inacio LGB, Molenzani AO. Investigating accessibility in Latin American science museums and centers. An Acad Bras Cienc [Internet]. 2020 Apr 17;92(1):e20191156. Available from: <http://dx.doi.org/10.1590/0001-3765202020191156>

55. Remillard ET, Campbell ML, Koon LM, Rogers WA. Transportation challenges for persons aging with mobility disability: Qualitative insights and policy implications. Disabil Health J [Internet]. 2022 Jan;15(1S):101209. Available from: <http://dx.doi.org/10.1016/j.dhjo.2021.101209>

56. Eisenberg Y, Heider A, Gould R, Jones R. Are communities in the United States planning for pedestrians with disabilities? Findings from a systematic evaluation of local government barrier removal plans. Cities [Internet]. 2020 Jul 1;102:102720. Available from: <http://dx.doi.org/10.1016/j.cities.2020.102720>

57. Powell RM. Confronting Eugenics Means Finally Confronting Its Ableist Roots. William & Mary Journal of Race, Gender, and Social Justice [Internet]. 2021 [cited 2023 Nov 22];27(3):607. Available from: <https://scholarship.law.wm.edu/wmjowl/vol27/iss3/2>

58. McConnell D, Phelan S. The devolution of eugenic practices: Sexual and reproductive health and oppression of people with intellectual disability. Soc Sci Med [Internet]. 2022 Apr;298:114877. Available from: <http://dx.doi.org/10.1016/j.socscimed.2022.114877>

59. Burke CS, Castaneda CJ. The public and private history of eugenics: an introduction. Public Hist [Internet]. 2007 Summer;29(3):5–17. Available from: <http://dx.doi.org/10.1525/tph.2007.29.3.5>

60. Iredale R. Eugenics and its relevance to contemporary health care. Nurs Ethics [Internet]. 2000 May;7(3):205–14. Available from: <http://dx.doi.org/10.1177/096973300000700303>

61. Lau PL. The Legacy of Eugenics in Contemporary Law. In: Lau PL, editor. Comparative Legal Frameworks for Pre-Implantation Embryonic Genetic Interventions [Internet]. Cham: Springer International Publishing; 2019. p. 27–72. Available from: <https://doi.org/10.1007/978-3-030-22308-3_2>

62. Sanchez-Rivera R. From preventive eugenics to slippery eugenics: Population control and contemporary sterilisations targeted to indigenous peoples in Mexico. Sociol Health Illn [Internet]. 2023 Jan;45(1):128–44. Available from: <http://dx.doi.org/10.1111/1467-9566.13556>

63. Radi B. Reproductive injustice, trans rights, and eugenics. Sex Reprod Health Matters [Internet]. 2020 Dec;28(1):1824318. Available from: <http://dx.doi.org/10.1080/26410397.2020.1824318>

64. Kaplan K, Brusilovskiy E, O’Shea AM, Salzer MS. Child Protective Service Disparities and Serious Mental Illnesses: Results From a National Survey. Psychiatr Serv [Internet]. 2019 Mar 1;70(3):202–8. Available from: <http://dx.doi.org/10.1176/appi.ps.201800277>

65. Appleman LI. Deviancy, Dependency, and Disability: the Forgotten History of Eugenics and Mass Incarceration. Duke Law J [Internet]. 2018 Dec;68(3):417–78. Available from: <https://scholarship.law.duke.edu/dlj/vol68/iss3/1>

66. Chow WS, Priebe S. Understanding psychiatric institutionalization: a conceptual review. BMC Psychiatry [Internet]. 2013 Jun 18;13:169. Available from: <http://dx.doi.org/10.1186/1471-244X-13-169>

67. Cheeseman NA. Understanding the History of Institutionalization: Making connections to De-institutionalization and the Olmstead Act for Persons with Intellectual Disabilities in the State of Illinois [Internet]. National Louis University; 2015 [cited 2023 Sep 29]. Available from: <https://digitalcommons.nl.edu/diss/127>

68. Stangis G. “Places of Such Towering Misery”: The History of the Institutionalization of Disabled People and Deinstitutionalization [Internet]. Oakland University; 2022. Available from: <http://hdl.handle.net/10323/11469>

69. Kelsey P. Disability and Native North American Boarding School Narratives: Madonna Swan and Sioux Sanitorium. Journal of Literary & Cultural Disability Studies [Internet]. 2013 [cited 2023 Sep 29];7(2):195–211. Available from: <http://dx.doi.org/10.3828/jlcds.2013.14>

70. Crenshaw KW. Demarginalizing the Intersection of Race and Sex: A Black Feminist Critique of Antidiscrimination Doctrine, Feminist Theory and Antiracist Politics. Univ Chic Leg Forum [Internet]. 1989 [cited 2023 Jul 18];1989:139. Available from: <https://scholarship.law.columbia.edu/faculty_scholarship/3007>

71. Collins PH. Black feminist thought: Knowledge, consciousness, and the politics of empowerment. 30th anniversary edition. London, England: Routledge; 2022.

72. Combahee River Collective. The Combahee River Collective Statement [Internet]. 1978 [cited 2023 Sep 29]. Available from: <https://americanstudies.yale.edu/sites/default/files/files/Keyword%20Coalition_Readings.pdf>

73. Bowleg L. The problem with the phrase women and minorities: intersectionality-an important theoretical framework for public health. Am J Public Health [Internet]. 2012 Jul;102(7):1267–73. Available from: <http://dx.doi.org/10.2105/AJPH.2012.300750>

74. Sins Invalid. Skin, tooth, and bone: The basis of movement is our people: A disability justice primer [Internet]. 2019. Available from: <https://www.sinsinvalid.org/disability-justice-primer>

75. Chen MY, Kafer A, Kim E, Minich JA. Crip Genealogies [Internet]. Duke University Press; 2023. 365 p. Available from: <http://dx.doi.org/10.1215/9781478023852>

76. Hinton A. On Fits, Starts, and Entry Points: The Rise of Black Disability Studies. caj [Internet]. 2021 [cited 2023 Oct 4];64(1):11–29. Available from: <https://muse.jhu.edu/article/787291>

77. Schalk S, Kim JB. Integrating Race, Transforming Feminist Disability Studies. Signs: Journal of Women in Culture and Society [Internet]. 2020 Sep 1;46(1):31–55. Available from: <https://doi.org/10.1086/709213>

78. Levine A, Breshears B. Discrimination at every turn: An intersectional ecological lens for rehabilitation. Rehabil Psychol [Internet]. 2019 May;64(2):146–53. Available from: <http://dx.doi.org/10.1037/rep0000266>

79. Carter MKA, McGill LS, Aaron RV, Hosey MM, Keatley E, Sanchez Gonzalez ML. We still cannot breathe: Applying intersectional ecological model to COVID-19 survivorship. Rehabil Psychol [Internet]. 2023 May;68(2):112–20. Available from: <http://dx.doi.org/10.1037/rep0000495>

80. Ferri BA, Connor DJ. Tools of Exclusion: Race, Disability, and (Re)segregated Education. Teach Coll Rec [Internet]. 2005 Mar 1;107(3):453–74. Available from: <https://doi.org/10.1111/j.1467-9620.2005.00483.x>

81. Ineese-Nash N. Disability as a Colonial Construct: The Missing Discourse of Culture in Conceptualizations of Disabled Indigenous Children. CJDS [Internet]. 2020 Sep 26 [cited 2023 Oct 4];9(3):28–51. Available from: <http://dx.doi.org/10.15353/cjds.v9i3.645>

82. Wickenden M. Disability and other identities?-how do they intersect? Front Rehabil Sci [Internet]. 2023 Aug 10;4:1200386. Available from: <http://dx.doi.org/10.3389/fresc.2023.1200386>

83. Mallipeddi NV, VanDaalen RA. Intersectionality Within Critical Autism Studies: A Narrative Review. Autism Adulthood [Internet]. 2022 Dec 1;4(4):281–9. Available from: <http://dx.doi.org/10.1089/aut.2021.0014>

84. Maroney MR, Horne SG. “Tuned into a different channel”: Autistic transgender adults’ experiences of intersectional stigma. J Couns Psychol [Internet]. 2022 Nov;69(6):761–74. Available from: <http://dx.doi.org/10.1037/cou0000639>

85. Wallisch A, Boyd BA, Hall JP, Kurth NK, Streed CG Jr, Mulcahy A, et al. Health Care Disparities Among Autistic LGBTQ+ People. Autism Adulthood [Internet]. 2023 Jun 1;5(2):165–74. Available from: <http://dx.doi.org/10.1089/aut.2022.0006>

86. Rivas Velarde M. Indigenous Perspectives on Disability. In: The Oxford Handbook of the Sociology of Disability [Internet]. 2021 [cited 2023 Sep 29]. p. 566–82. Available from: <https://doi.org/10.1093/oxfordhb/9780190093167.001.0001>

87. Kushalnagar P, Miller CA. Health Disparities Among Mid-to-Older Deaf LGBTQ Adults Compared with Mid-to-Older Deaf Non-LGBTQ Adults in the United States. Health Equity [Internet]. 2019 Oct 30;3(1):541–7. Available from: <http://dx.doi.org/10.1089/heq.2019.0009>

88. Gendron T, Camp A, Amateau G, Mullen M, Jacobs K, Inker J, et al. The next critical turn for ageism research: The intersections of ageism and ableism. Gerontologist [Internet]. 2023 Jun 2; Available from: <https://doi.org/10.1093/geront/gnad062>

89. Nguyen TV, King J, Edwards N, Dunne MP. “Under great anxiety”: Pregnancy experiences of Vietnamese women with physical disabilities seen through an intersectional lens. Soc Sci Med [Internet]. 2021 Sep;284:114231. Available from: <http://dx.doi.org/10.1016/j.socscimed.2021.114231>

90. Griffin M, Bailey KA, Lopez KJ. #BodyPositive? A critical exploration of the body positive movement within physical cultures taking an intersectionality approach. Front Sports Act Living [Internet]. 2022 Oct 10;4:908580. Available from: <http://dx.doi.org/10.3389/fspor.2022.908580>

91. Subih M. The Intersectionality of Disability, Religion, Ethnicity, and Gender: Muslim Arabs in the United States [Internet]. Eisenman L, editor. [Ann Arbor, United States]: University of Delaware; 2023. Available from: <https://www.proquest.com/docview/2832647486>

92. Atkin K, Hussain Y. Disability and Ethnicity: how young Asian disabled people make sense of their lives. In: Disability, culture and identity [Internet]. Routledge; 2014. p. 161–79. Available from: <https://doi.org/10.4324/9781315847634>

93. Hassiotis A. The Intersectionality of Ethnicity/race and Intellectual and Developmental Disabilities: Impact on Health Profiles, Service Access and Mortality. J Ment Health Res Intellect Disabil [Internet]. 2020 Jul 2;13(3):171–3. Available from: <https://doi.org/10.1080/19315864.2020.1790702>

94. Pisani M, Grech S, Mostafa A. Disability and Forced Migration: Intersections and Critical Debates. In: Grech S, Soldatic K, editors. Disability in the Global South: The Critical Handbook [Internet]. Cham: Springer International Publishing; 2016. p. 285–301. Available from: <https://doi.org/10.1007/978-3-319-42488-0_18>

95. Stienstra D, Nyerere L. Race, Ethnicity and Disability: Charting Complex and Intersectional Terrains. In: Grech S, Soldatic K, editors. Disability in the Global South: The Critical Handbook [Internet]. Cham: Springer International Publishing; 2016. p. 255–68. Available from: <https://doi.org/10.1007/978-3-319-42488-0_16>
